# Risk Factors Associated with Bruises in Beef Cattle Carcasses

**DOI:** 10.3390/ani15172608

**Published:** 2025-09-05

**Authors:** Fabio Martins Guerra Nunes Dias, Fredson Vieira e Silva, André Guimarães Maciel e Silva, Jonas Carneiro Araújo, Guilherme Jordão de Magalhães Rosa, José Bento Sterman Ferraz

**Affiliations:** 1 College of Animal Sciences and Food Engineering, University of São Paulo, Pirassununga 13635-900, SP, Brazil; jbferraz@usp.br; 2 Department of Agricultural Sciences, State University of Montes Claros, Janaúba Campus, Janaúba 39448-581, MG, Brazil; fredson.silva@unimontes.br; 3 Institute of Veterinary Medicine, Federal University of Pará, Castanhal Campus, Castanhal 68741-740, PA, Brazil; andregms@ufpa.br; 4 Department of Animal Health and Production, Federal Rural University of Amazônia, Belém Campus, Belém 66077-580, PA, Brazil; carneirojonas6@gmail.com; 5 Department of Animal and Dairy Sciences, University of Wisconsin-Madison, Madison, WI 53706, USA; grosa@wisc.edu

**Keywords:** animal welfare, beef carcasses, bruising

## Abstract

Bruises in beef cattle carcasses are important indicators related to pre-slaughter handling and transport conditions, with implications for animal welfare and meat quality. This study analysed 19.4 million cattle carcasses transported from 42,805 farms to 38 slaughterhouses in Brazil to identify factors associated with bruising. Logistic regression models were used to assess the effects of sex, age, transport distance from farm to industry, and truck class. At least one bruise was found in 33.8% of the analysed carcasses. Older animals had a greater prevalence of bruising, and females were the most affected. The relationship between transport distance and bruising varied across carcass regions, showing distinct patterns rather than a uniform trend. Compared with larger-capacity vehicles, smaller trucks increased the risk of bruising. The round, rump, and flank regions presented the greatest number of bruises. The models assessing individual effects demonstrated good overall performance, with accuracy ranging from 75% to 82% in identifying bruises. The best performance was observed for round-rump, likely due to the higher frequency of bruises in these cuts. These findings highlight the need to improve transport logistics, adopt better handling practices, and implement specific interventions to reduce bruising.

## Simple Summary

This study analysed 19.4 million beef cattle carcasses in Brazil. Bruising was found in 33.8% of carcasses. Older animals and females were the most affected, and smaller trucks increased the risk. These findings highlight the need for improved transport logistics and handling practices. Producers, industry stakeholders, and policymakers can use these insights to minimize economic losses, improve meat quality, and enhance animal welfare.

## 1. Introduction

The cattle herd in Brazil reached a total of 239 million heads in 2023 [1], making it one of the largest commercial herds for beef production in the world. However, the growth of this sector presents challenges related to logistics and management conditions. One of the most critical aspects is the presence of bruises on carcasses, which are often associated with intrinsic and extrinsic factors, such as animal characteristics and transport conditions [2,3]. Bruises on cattle carcasses are widely recognized as potential welfare indicators, reflecting the impact of conditions experienced during the pre-slaughter stages [4,5]. Additionally, they compromise the commercial value of carcasses, require processing interventions and consequently affect the efficiency of the production system [6,7].

Although studies have suggested that variables such as age, sex, truck type, and transport distance influence the frequency of bruising on cattle carcasses [3,8,9,10], uncertainties remain regarding which factors have the greatest impact and how they interact. Identifying the causes of bruises in carcasses contributes to the development of strategies to improve animal welfare during land transportation [4,11]. Moreover, specific primal cuts, such as the rump, sometimes grouped with the round in previous analyses, may exhibit unique bruise patterns. These cuts have commercial relevance in the meat market [12].

Given this context, this study aimed to identify risk factors associated with the occurrence of bruises in beef cattle carcasses based on a large dataset covering animal characteristics, transport conditions, and slaughter logistics.

## 2. Materials and Methods

### 2.1. Data Description

The analysed dataset covers three years (January 2018 to December 2020) of beef cattle slaughter data from 10 Brazilian states (federative units), which represent a portion of the country’s 26 states. A total of 19.4 million beef cattle carcasses were evaluated in the study, after excluding 2.91% of the original records due to data inconsistencies to ensure the reliability of the analysed information. This number of carcasses corresponds to 21.54% of the animals slaughtered in Brazil between 2018 and 2020 [13].

The states included in this study, with percentages indicating the proportion of carcasses analysed in each state, were Acre (2.1%), Bahia (1.6%), Goiás (13.1%), Minas Gerais (5.9%), Mato Grosso do Sul (21.1%), Mato Grosso (29.9%), Pará (6.3%), Rondônia (10.8%), São Paulo (5.8%), and Tocantins (3.4%). This dataset contains comprehensive information on cattle transportation, originating from 42,805 farms and processed at 38 slaughterhouses. The data reflect real-world information on both transport logistics and carcass characteristics, and were provided by the packing plant company responsible for managing the logistics of transporting the animals from farms to slaughterhouses.

### 2.2. Bruise Identification

**Figure 1:**
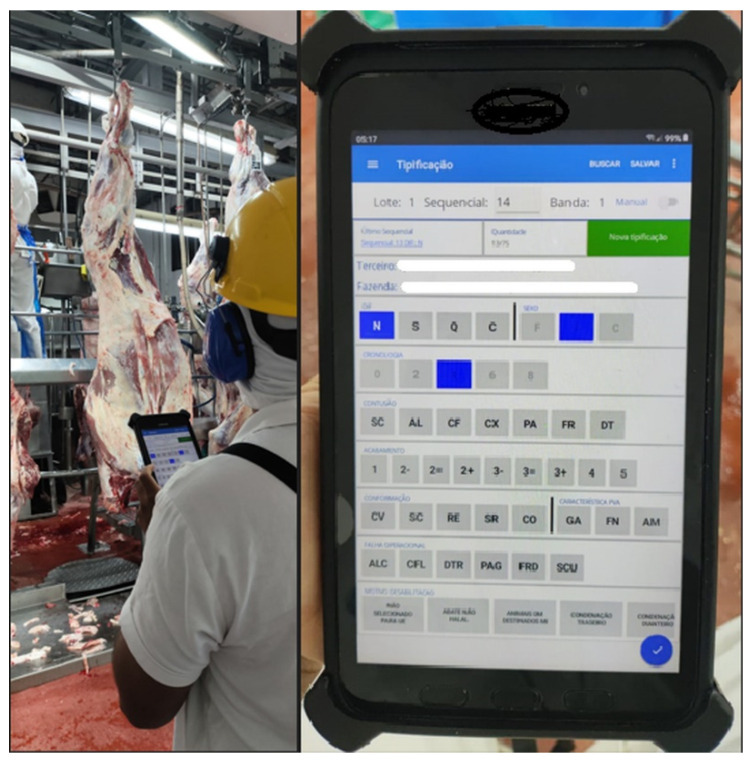
Carcass grading process in beef cattle: a trained technician evaluates hot carcasses for bruises (**left**), using a digital grading system on a tablet (**right**).

The identification of bruises was performed by a trained technician from the beef processing company who has undergone annual training and certification (Figure 1). The assessment follows a daily verification process, ensuring a 95% accuracy rate. Each hot carcass was evaluated for bruises in the primal cuts immediately before the weighing stage, after the carcass had undergone the trimming process. Both hot half-carcasses were evaluated individually.

**Figure 2:**
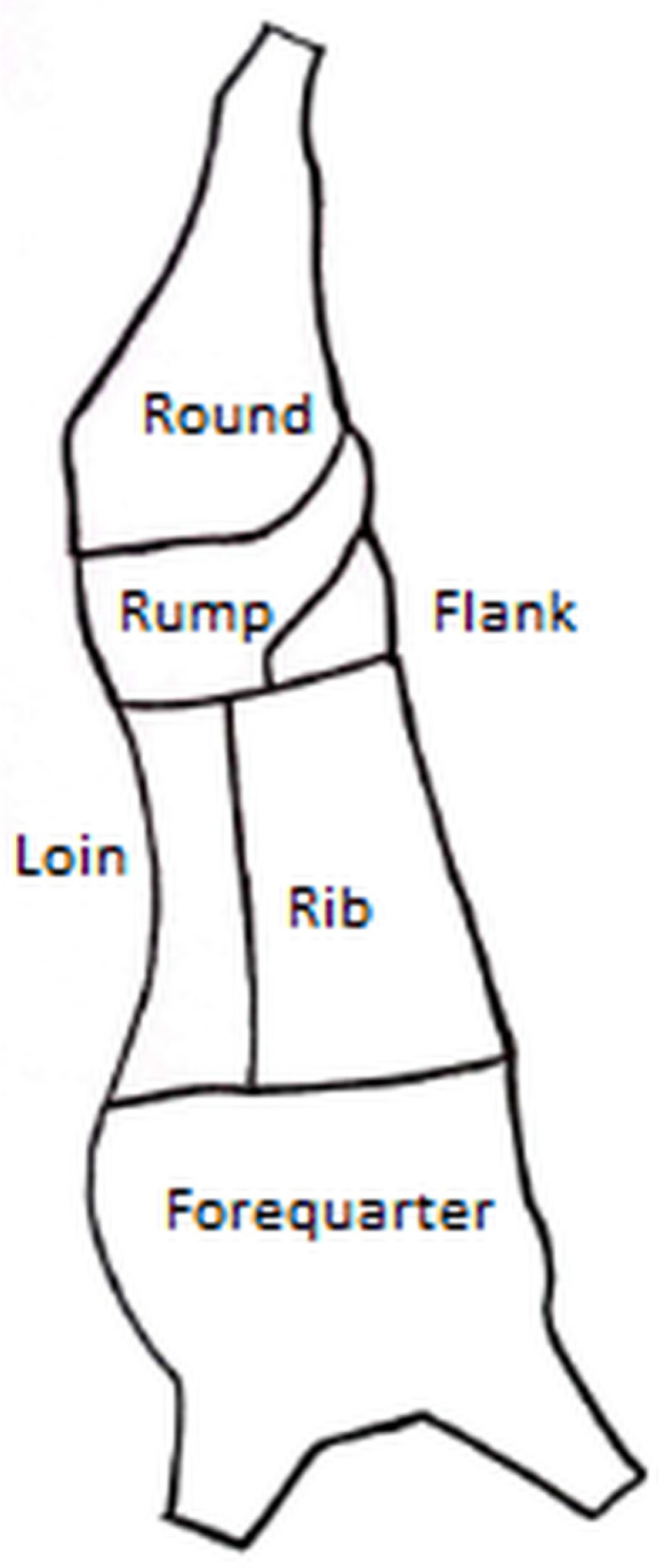
Half-carcass of beef showing the divisions of primal cuts.

Bruises were identified regardless of their size, color, or number of injuries present. The primal cuts evaluated included round, rump, loin, flank, rib, and forequarter (Figure 2) [14]. For this analysis, a primal cut was recorded as having bruises if at least one bruise was detected on either side or both sides of the cut. Bruises in the primal cuts were analysed on the basis of the following predictive variables: distance travelled by the truck between the origin farm and the destination slaughterhouse, truck class, animal age and sex, and the subcutaneous fat score of the carcasses.

### 2.3. Distance Categories in Cattle Transport

The classification of distances from the farm to the slaughterhouse, considering all truck classes, was based on the 10th, 25th, 50th, 75th, and 90th percentiles. The categories were defined as follows: ‘very short’ for distances up to 50 km, ‘short’ for distances from 51 to 90 km, ‘medium’ for distances from 91 to 160 km, ‘medium-long’ for distances from 161 to 270 km, ‘long’ for distances from 271 to 399 km, and ‘very long’ for distances of 400 km or more.

### 2.4. Truck Class

**Figure 3:**
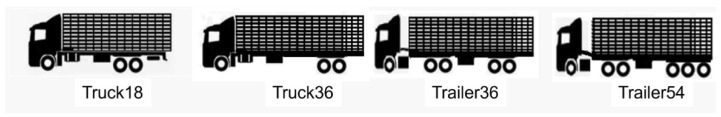
Representative truck classes used for cattle transportation in this study, categorized by type of traction and live load capacity: Truck18 (capacity for 18 animals), Truck36 (capacity for 36 animals), Trailer36 (capacity for 36 animals), and Trailer54 (capacity for 54 animals).

The freight orders presented more than 600 types of vehicles or combinations of different vehicles in the transportation of larger lots. To provide an overview of cattle transportation from farms to slaughterhouses, the vehicles listed in the freight orders were grouped according to the type of traction and their live load capacity, forming truck classes. The types of traction used were categorized as vehicles pulled by tractor units (trailers) or rigid trucks (trucks). Vehicles were categorized into five groups: Truck18 (capacity of 18 animals), Truck36 (36 animals), Trailer36 (36 animals), and Trailer54 (54 animals), which are the four main truck classes illustrated in Figure 3; and Mixed, which includes freight orders composed of combinations of different truck classes due to their heterogeneous nature.

### 2.5. Classification by Age and Sex

The age class was based on dentition (PIT = permanent incisor teeth) as follows: 0-PIT for animals ≤19 months, 2-PIT for 20–26 months, 4-PIT for 27–36 months, 6-PIT for 37–63 months, and 8-PIT for animals older than 63 months. Animals were categorized by sex into three groups for analysis: beef bulls (non-castrated males), steers (castrated males), and females. Both age and sex classifications followed the Brazilian beef carcass grading system [15].

### 2.6. Subcutaneous Fat Score Classification of Carcasses

The subcutaneous fat score of the carcasses was evaluated by a trained grader according to the methodology adopted by the slaughterhouse company, which is aligned with the Brazilian carcass grading system [15]. The evaluation used five fat score levels, categorized as follows: 1—absent fat, 2—scarce fat, 3—medium fat, 4—uniform fat, and 5—excessive fat. Since no carcasses with a fat score of 5 and only a few with a fat score of 4 (326,988) were found in the database, scores 3 and 4 were grouped into a single category to avoid compromising the analysis.

### 2.7. Statistical Analysis of Data

All analyses were performed using R (version 4.4.3) in RStudio.

#### 2.7.1. Descriptive Analysis

Bruises from each carcass of animals slaughtered were analysed, considering six primal cuts: rump, loin, round, forequarter, flank, and rib. Bruises were categorized into binary indicators for each cut and a global variable for carcasses with at least one bruise. The same approach was applied to the analysed variables, including the distance travelled from the farm to the slaughterhouse, truck class, animal age and sex, and subcutaneous fat thickness of the carcasses. The frequency of bruises and their associations with the analysed variables were calculated and expressed as percentages. Statistical analyses were performed via the data.table package.

#### 2.7.2. Least Absolute Shrinkage and Selection Operator (LASSO) Logistic Regression

The analysis was conducted via least absolute shrinkage and selection operator (LASSO) logistic regression to identify the main predictors of bruises in bovine carcasses across different primal cuts. Preliminary tests (mean adjusted generalized variance inflation factor) indicated an increase in multicollinearity among predictor variables as more variables were added to the models. Owing to this multicollinearity, LASSO was chosen because it performs variable selection and reduces model complexity. The response variable was the presence of bruises in a specific carcass cut. The predictor variables included the age and sex of the animals, the distance travelled from the farm to the slaughterhouse, the truck class used, and the subcutaneous fat cover of the carcasses.

The data were standardized, and a predictor matrix was created for model fitting. To address the imbalance in the response variable, weights were applied proportionally to the relative frequency of the classes. The model was adjusted via five-fold cross-validation (k = 5) to determine the optimal penalization parameter (lambda.min). Performance was evaluated via the area under the receiver operating characteristic (ROC) curve (AUC), and the importance of the variables was quantified via global odds ratios. Additionally, *p*-values were calculated for the estimated coefficients, allowing for the statistical assessment of the significance of the predictors.

#### 2.7.3. Logistic Models

Logistic models were fitted for each predictor variable, with the presence of carcass bruising as the response variable. Model performance was assessed via the odds ratio and AUC. To optimize the analysis and reduce data fragmentation, primary cuts were grouped according to their anatomical location, clustering adjacent regions to provide a more consistent assessment of bruising. A random effect for Farm was included in the models to account for potential variability among farms related to management practices and infrastructure, without aiming to evaluate specific farm effects individually. Preliminary tests indicated that slaughterhouse had no significant effect on bruising prevalence and was therefore not included in the final models. Additionally, analyses considering the effect of State are presented in the Supplementary Materials.

## 3. Results

### 3.1. Descriptive Analysis of the Prevalence of Carcass Bruises

#### 3.1.1. Prevalence of Bruises in Beef Carcasses and Distribution Across Primal Cuts

Among the more than 19.4 million carcasses studied, over 6,6 million carcasses (33.8%) showed bruises (Table 1). Among the primal cuts, round and flank had the highest percentages (overall average of 16.3%), followed by rump (14.3%), while the lowest values were recorded for forequarter and loin. The distribution of carcasses with at least one bruise across Brazilian states is presented in Table S1 and Figure S1.

#### 3.1.2. Journey Distance

With respect to the distance of the journey (Table 2), the percentage of bruises increased up to medium-long journeys and decreased over longer distances, ranging from 31.6% in very short transports to 34.5% in very long journeys. In the primal cuts, the lowest percentages occurred in very short transports, whereas the highest rates were observed in very long journeys for loins, flanks, and ribs. Forequarters consistently presented the lowest values across all distances.

#### 3.1.3. Class of Trucks

When analysing bruises by truck type (Table 3), Truck18 had the highest percentage of carcasses with bruises (42.2%), whereas Trailer54 had the lowest percentage of carcass bruises (26.9%). The Mixed class presented a low proportion of carcass bruises (28.9%). The Trailer54 and Mixed trucks, which generally consisted of Trailer54 trucks, presented a lower percentage of injuries in the hindquarter regions of the carcasses (round: 10.1, rump: 9.6 for Trailer54; round: 12.1, rump: 11.1 for Mixed), whereas the Truck18 trucks presented a greater percentage of carcass injuries, as well as a greater number of injuries in the primal cuts of the hindquarter (round: 24.07, rump: 19.7).

#### 3.1.4. Age of the Animals

The percentage of bruises increased across all primary cuts as the animals grew older (Table 4). Younger animals had lower percentages of bruises in the forequarter, round, and loin regions, with the highest percentage of bruises occurring in the flank area. In contrast, older animals presented the highest percentage of bruises in the round region. The lowest values were observed in the 0-PIT class (22.7%), whereas the 8-PIT class presented the highest percentage (54.6%). Among the primal cuts, the percentage of bruises also increased with age, with round, rump, and flank standing out for recording the highest values in the 8-PIT class. Forequarters consistently had the lowest percentages across all age classes, showing a gradual increase with age.

#### 3.1.5. Sex of the Animals

The percentage of bruises observed by the sex of the transported animals (Table 5) was greater in females (51.8%) and lower in beef bulls (24.3%). Castrated animals presented a greater percentage of bruises (32.7%) than beef bulls did. Among the primal cuts, round, rump, and flank were the most affected in females, with round and flank standing out, accounting for 28% of the total. Forequarters presented the lowest percentages across all categories, with a maximum value of 10.4% in females and a minimum of 4.8% in beef bulls.

#### 3.1.6. Subcutaneous Fat Score of Carcasses

Animals with a fat score of 1 had the lowest percentage of bruises (26.9%), whereas those with fat scores of 2 and 3 presented a higher percentage of bruising (FS-2: 33.83%; FS-3: 34.54%) (Table 6). Among all of the primal cuts, those with a score of 1 had the lowest percentage of bruises.

### 3.2. Odds Ratios and Performance of Least Absolute Shrinkage and Selection Operator (LASSO) Models

The models adjusted for the different primal cuts showed variable performance (Table 7). The best result was observed for round-rump (AUC = 0.72), with age and sex as the main predictors. For loin (AUC = 0.65), the highest odds ratios were associated with fat score, age, and sex. For flank-rib-forequarters (AUC = 0.67), the main predictors were age, sex, and fat score.

### 3.3. Logistic Regression Models with Individual Predictors

The models with individual effects showed good overall performance, with AUCs ranging from 0.75 to 0.82.

#### 3.3.1. Distance of the Journey

The analysis of the effects of transport distance on the occurrence of carcass bruises revealed distinct patterns among the primal cuts (Table 8). The odds ratios were higher for short distances in the round-rump, whereas in the loin and flank-rib-forequarter cuts, the values were lower than the reference (very long transport).

#### 3.3.2. Truck Classes

The analysis by truck class revealed variations among the primal cuts (Table 9). In round-rump, all truck classes had odds ratios lower than 1 compared to the reference group (Truck18). In loin, all classes presented odds ratios close to 1. For flank-rib-forequarter, Truck36 had the highest odds ratio, exceeding the reference group.

#### 3.3.3. Age Classes

The effects of age classes showed an increasing trend in the odds ratios as age increased (Table 10). The round-rump pattern exhibited the greatest variation, with odds rising as age increased.

#### 3.3.4. Animal Sex

The analyses by sex revealed lower odds ratios for beef bulls, followed by steers, in all cuts (Table 11).

#### 3.3.5. Subcutaneous Fat Score

The analysis of the subcutaneous fat score revealed lower odds ratios for Fat 1 than for the reference group (Fat 3) (Table 12). This pattern was observed across all primal cuts.

## 4. Discussion

The high proportion of cattle carcasses with bruises (33.8%) highlights a significant animal welfare issue and compromises product quality. Although the frequency of bruising in carcasses varies significantly between regions, studies have shown that the observed rates remain high, exceeding 37% [16,17,18,19]. The absence of a temporal analysis of bruises, as performed in other studies [20], prevents an exact determination of when bruises occurred—whether during pre-slaughter handling or in earlier stages. However, the variability observed in the data highlights the effects of the analysed variables on the prevalence of bruises.

While similar studies indicate that bruises occur more frequently in the hindquarters of the animal [16,21], others have reported a higher prevalence in the ribs or forequarters [22,23]. These authors suggest that adjustments in the environment, such as guillotine-style doors, should be considered to mitigate this problem. Environmental adjustments can facilitate animal movement and reduce the use of aversive handling methods [24]. The location of bruises may indicate possible causes of injuries, helping to identify critical points in handling that require intervention, given that there is no defined pattern for the prevalence of bruises. This aspect can be explored in experiments with a relatively high level of control to validate mitigation strategies.

As observed in this study, the loin primal cut has the lowest prevalence of bruises [22], but there are exceptions, as reported by Polizel Neto et al. (2015) [25]. This lower prevalence may be related to its anatomical position, which offers greater protection and reduces exposure to direct impacts. On the other hand, it is suggested that the higher prevalence of bruises in the hindquarters is associated with the dynamics of cattle movement. While the balance point is located near the shoulders, guiding the movement direction [26], the driving force comes from the hind limbs, which are responsible for propulsion [27]. This mismatch between control and locomotion, combined with fatigue and animal age [28], may increase susceptibility to impacts and, consequently, the prevalence of bruises in this region.

Considering that the LASSO models involving the primal cuts, the factors with the greatest influence on the occurrence of bruising are sex and age. The best performance observed in the round-rump model (AUC = 0.72) may be related to the higher frequency of bruises in this cut, resulting in a more robust representation of response variable patterns. On the other hand, the loin model (AUC = 0.65) has a lower discrimination capacity because of the lower prevalence of bruises in this cut and lower data variability. Despite the moderate AUC (0.65–0.72), the goal was not to predict bruising, but to reduce multicollinearity and identify key variables to support more effective handling and transport strategies, enabling targeted preventive measures.

The single-predictor models performed better, mainly due to the inclusion of farms as a random effect, which accounted for local structure and management variability, allowing greater focus on the studied predictors. Consistently, females have higher odds of presenting bruises than males across all primal cut groups. Compared with beef bulls, females had twice as many bruises, and their likelihood of bruising in the round-rump region was 335% higher. Females are repeatedly identified as the sex most prone to bruising [17,29,30]. Several explanations exist: the reticular layer tends to be thicker in males as they age [31], and heifers exhibit significantly higher temperament scores than steers do [32], characteristics that may impact the prevalence of injuries.

Animal age proved to be one of the most relevant factors, with bruises increasing significantly in older animals (eight permanent teeth) compared with younger ones (zero permanent teeth). This trend was most evident in the round-rump cuts, where the probability of bruising was 75% lower in zero-tooth animals than in older ones. Previous studies have also highlighted age as a determining factor in the occurrence of bruises in cattle [33,34].

The data indicate that animals with lower subcutaneous fat deposition have fewer bruises than those with greater fat cover. This may be related to greater muscle firmness and tissue cohesion in leaner animals, as lean cattle have higher concentrations of cross-links and proteoglycans in intramuscular connective tissue, which increases muscle resistance [35]. Additionally, the presence of decorin and tenascin-X, extracellular matrix molecules associated with muscle structure, contributes to muscle firmness [36], which may influence the response to impacts during handling and transport. Furthermore, a confounding effect related to animal category may exist, as males tend to be younger, have a greater adult weight (later-maturing) [37] when compared to females, and consequently accumulate less fat. Thus, the observed pattern may reflect not only the impact of subcutaneous fat, but also the physiological and behavioral differences between sex categories.

Least absolute shrinkage and selection operator logistic regression analysis indicated that transport distance had a significant effect on bruising in the round-rump and loin regions. Conflicting reports exist in the literature regarding the effect of transport time on carcass bruises. While some studies did not identify a significant relationship between these variables [17,19], others reported a positive influence of distance on the number of bruises [2,30]. The incidence of bruising in the round-rump region increased in short- and medium-distance transports, peaking at 160 km (OR = 1.44). However, for longer journeys (≥270 km), the risk declined, reaching its lowest level in transports between 270 and 399 km (OR = 0.83). This suggests that bruising in this region is more associated with handling and instability in the initial stages of transport, rather than with prolonged travel. Longer journeys generally involve more suitable vehicles and take place mostly on paved roads, with unpaved sections limited to farm access, providing a more stable environment that reduces the risk of bruising. Moreover, older animals and females—more vulnerable to bruising—are less frequently transported over long distances [38], which may further explain the lower incidence of bruising in extended transports.

In contrast, loin and flank, rib, and forequarter regions showed the opposite pattern, with bruising increasing as transport distance increased. This suggests that the causes of bruising differ between body regions. In these areas, injuries seem to result from prolonged physical contact between animals and the truck structure, as well as the effects of fatigue during extended journeys. As animals become more exhausted, they may struggle to maintain balance, increasing the risk of bruising from repeated pressure and friction [39].

Least Absolute Shrinkage and Selection Operator regression models apply a penalty that reduces the coefficients of variables with lesser impact, favoring those that better explain data variability. In the models, truck class had a modest effect, suggesting that while the effect is not strong, some influence may exist. This result may be explained by the fact that intrinsic animal variables, such as sex, age, and fat score, have greater explanatory power, capturing much of the variation in bruise risk. Nevertheless, smaller trucks, such as Truck18, had a greater prevalence of bruised carcasses than did larger-capacity vehicles, such as Trailer54, particularly in the round and rump regions. The likelihood of bruising in this region was 16% lower for Trailer54 trucks than for Truck18 trucks. Conflicting findings have been reported in the literature [3,20]. However, smaller trucks are often used in smaller rural properties, which tend to have lower-quality infrastructure, potentially influencing injury prevalence during transport [21,24]. Furthermore, females are commonly transported in smaller vehicles, which may have influenced the observed results.

## 5. Conclusions

The high prevalence of bruises in cattle carcasses raises concerns about animal welfare and meat quality. The results demonstrate that intrinsic factors, such as sex and age, strongly influence the occurrence of bruises, with a higher prevalence in females and older animals. Additionally, smaller trucks increased the incidence of bruising, especially in hindquarter cuts, whereas higher-capacity vehicles helped mitigate these effects. Furthermore, the occurrence of bruises varied across different carcass regions depending on transport distance, reinforcing the need for targeted strategies to minimize injuries.

## Figures and Tables

**Table 1:** Number and percentage of beef cattle carcasses with at least one bruise.

Animal Count	Bruised Carcasses (%)	Bruised Primal Cut (%) ^1^					
		Round	Rump	Loin	Flank	Rib	Forequarter
19,403,384	33.84	16.31	14.27	8.24	16.30	9.94	6.68

^1^ Percentages of bruised primal cuts are not mutually exclusive, as a single carcass may present bruises in more than one cut.

**Table 2:** Number and percentage of beef cattle carcasses with at least one bruise in primal cuts, categorized by journey distance class.

Journey Distance ^1^	Animal Count	Bruised Carcasses (%)	Bruised Primal Cut (%) ^2^					
			Round	Rump	Loin	Flank	Rib	Forequarter
Very short	1,886,025	31.58	12.94	13.15	7.10	14.68	8.90	6.29
Short	3,003,919	33.19	16.07	13.74	7.28	15.23	9.85	6.64
Medium	4,440,232	36.01	18.75	15.87	7.94	16.30	10.01	6.99
Medium-long	5,221,022	33.33	16.28	13.48	8.07	16.39	9.56	6.42
Long	2,980,205	33.19	16.03	14.18	9.20	17.21	10.40	6.84
Very long	1,871,981	34.45	14.88	14.82	10.56	17.90	11.27	6.88

^1^ Very short for transports of up to 50 km; short for transports of up to 90 km; medium for transports of up to 160 km; medium-long for transports of up to 270 km; long for transports of up to 399 km; Very very long for transports from 400 km onwards. ^2^ Percentages of bruised primal cuts are not mutually exclusive, as a single carcass may present bruises in more than one cut.

**Table 3:** Number and percentage of beef cattle carcasses with at least one bruise in primal cuts, categorized by truck class.

Truck Class ^1^	Animal Count	Bruised Carcasses (%)	Bruised Primal Cut (%) ^2^					
			Round	Rump	Loin	Flank	Rib	Forequarter
Mixed	6,814,449	28.89	12.09	11.17	6.91	13.37	8.10	5.18
Trailer36	947,210	39.79	19.40	19.00	9.26	17.51	11.19	7.96
Trailer54	4,537,052	26.95	10.10	9.60	6.64	13.53	7.91	5.10
Truck18	6,997,942	42.29	24.07	19.69	10.42	20.73	12.87	8.97
Truck36	106,731	35.11	14.82	14.15	8.72	18.82	10.21	7.90

^1^ Mixed = more than one truck type per freight order; Trailer36, trailers with a load capacity of up to 36 animals; Trailer54, trailers with a load capacity of up to 54 animals; Truck18, rigid trucks with a load capacity of up to 18 animals; Truck36, rigid trucks with a load capacity of up to 36 animals. ^2^ Percentages of bruised primal cuts are not mutually exclusive, as a single carcass may present bruises in more than one cut.

**Table 4:** Number and percentage of beef cattle carcasses with at least one bruise in primal cuts, categorized by age class.

Age ^1^	Animal Count	Bruised Carcasses (%)	Bruised Primal Cut (%) ^2^					
			Round	Rump	Loin	Flank	Rib	Forequarter
0-PIT	2,623,684	22.69	5.88	7.89	5.29	11.51	6.44	4.06
2-PIT	5,341,551	26.92	9.56	10.34	6.34	12.99	7.45	4.62
4-PIT	4,823,549	30.15	13.47	12.05	7.17	14.02	8.53	5.61
6-PIT	2,994.906	36.80	19.76	16.18	8.82	16.84	10.65	7.48
8-PIT	3,619,694	54.58	34.78	26.09	14.10	27.22	17.43	12.38

Abbreviations: PIT = permanent incisor teeth. ^1^ Animals are categorized by dentition on the basis of age: ≤19 months = 0-PIT; 20–26 months = 2-PIT; 27–36 months = 4-PIT; 37–63 months = 6-PIT; and >63 months = 8-PIT. ^2^ Percentages of bruised primal cuts are not mutually exclusive, as a single carcass may present bruises in more than one cut.

**Table 5:** Number and percentage of beef cattle carcasses with at least one bruise in primal cuts, categorized by sex.

Sex	Animal Count	Bruised Carcasses (%)	Bruised Primal Cut (%) ^1^					
			Round	Rump	Loin	Flank	Rib	Forequarter
Beef bulls	11,149,508	24.27	10.14	9.31	5.80	9.99	7.21	4.78
Steers	2,179,662	32.72	15.09	14.31	8.49	16.28	8.37	5.98
Females	6,074,214	51.81	28.09	23.36	12.62	27.87	15.50	10.41

^1^ Percentages of bruised primal cuts are not mutually exclusive, as a single carcass may present bruises in more than one cut.

**Table 6:** Number and percentage of beef cattle carcasses with at least one bruise in primal cuts, categorized by fat score.

Fat Score ^1^	Animal Count	Bruised Carcasses (%)	Bruised Primal Cut (%) ^2^					
			Round	Rump	Loin	Flank	Rib	Forequarter
FS-1	791,265	26.89	14.96	11.82	4.29	10.01	7.52	5.12
FS-2	10,657,234	33.83	17.01	14.61	8.07	16.11	9.96	6.52
FS-3	7,954,885	34.54	15.52	14.06	8.85	17.17	10.15	7.05

Abbreviations: FS = fat score. ^1^ FS-1 = absent fat; FS-2 = scarce fat; FS-3 = medium fat. ^2^ Percentages of bruised primal cuts are not mutually exclusive, as a single carcass may present bruises in more than one cut.

**Table 7:** Global odds ratios and model metrics from least absolute shrinkage and selection operator logistic regression for predictors of bruising of carcasses in different primal cuts of cattle.

Predictor	Carcass						Primal Cut					
				Round and Rump			Loin			Flank, Rib, and Forequarter		
	Odds	LCI	UCI	Odds	LCI	UCI	Odds	LCI	UCI	Odds	LCI	UCI
Journey Distance	1.06	0.868	1.284	1.19	0.975	1.443	1.18	0.971	1.437	1.04	0.854	1.263
Truck class	1.00	0.822	1.217	1.01	0.831	1.230	1.01	0.830	1.229	1.04	0.858	1.269
Age	1.87	1.541	2.280	2.44	2.003	2.964	1.65	1.354	2.003	1.59	1.305	1.932
Sex	1.95	1.600	2.368	1.99	1.635	2.420	1.56	1.282	1.897	1.85	1.519	2.248
Fat score	1.17	0.962	1.424	1.04	0.857	1.269	1.77	1.452	2.149	1.24	1.018	1.507

Abbreviations: LCI = lower confidence interval; UCI = upper confidence interval; AUC = area under the receiver operating characteristic curve. Model performance metrics and *p*-value: round and rump model: AUC = 0.72; *p* < 0.001. Loin model: AUC = 0.65; *p* < 0.001. Flank, rib, and forequarter model: AUC = 0.67; *p* < 0.001.

**Table 8:** Odds ratios and model metrics from logistic regression for bruises in beef cattle carcasses by transport distance across different primal cuts.

Journey Distance ^1^	Round and Rump			Loin			Flank, Rib, and Forequarter		
	Odds	LCI	UCI	Odds	LCI	UCI	Odds	LCI	UCI
Very long (ref.)									
Intercept	0.28	0.277	0.287	0.11	0.109	0.113	0.36	0.351	0.362
Very short	1.38	1.361	1.398	0.69	0.666	0.690	0.77	0.756	0.776
Short	1.35	1.331	1.362	0.63	0.624	0.644	0.82	0.809	0.827
Medium	1.44	1.429	1.457	0.65	0.645	0.662	0.79	0.781	0.796
Medium-long	1.03	1.019	1.038	0.67	0.657	0.673	0.79	0.782	0.796
Long	0.83	0.820	0.835	0.81	0.803	0.822	0.83	0.822	0.836

Abbreviations: LCI = lower confidence interval; UCI = upper confidence interval; ref. = reference; AUC = area under the receiver operating characteristic curve. ^1^ Very short for transports of up to 50 km; short for transports of up to 90 km; medium for transports of up to 160 km; medium-long for transports of up to 270 km; long for transports of up to 399 km; very long for transports from 400 km onwards. Model performance metrics: AUC and *p*-value: round and rump model: AUC = 0.81; *p* < 0.001. Loin model: AUC = 0.76; *p* < 0.001. Flank, rib, and forequarter model: AUC = 0.77; *p* < 0.001.

**Table 9:** Odds ratios and model metrics from logistic regression for bruises in beef cattle carcasses by truck class during transport across different primal cuts.

Truck Class ^1^	Round and Rump			Loin			Flank, Rib, and Forequarter		
	Odds	LCI	UCI	Odds	LCI	UCI	Odds	LCI	UCI
Truck18 (ref.)									
Intercept	0.35	0.340	0.351	0.08	0.077	0.079	0.29	0.290	0.298
Mixed	0.88	0.875	0.883	0.99	0.982	0.994	0.94	0.935	0.943
Trailer36	0.96	0.953	0.966	1.01	0.999	1.019	0.95	0.940	0.953
Trailer54	0.84	0.833	0.841	1.04	1.035	1.050	0.98	0.970	0.979
Truck36	0.88	0.867	0.900	0.99	0.964	1.016	1.12	1.098	1.137

Abbreviations: LCI = lower confidence interval; UCI = upper confidence interval; ref. = reference; AUC = area under the receiver operating. ^1^ Mixed = more than one truck type per freight order; Trailer36: trailers with a load capacity of up to 36 animals; Trailer54: trailers with a load capacity of up to 54 animals; Truck18: rigid trucks with a load capacity of up to 18 animals; Truck36: rigid trucks with a load capacity of up to 36 animals. Model performance metrics: AUC and *p*-value: round and rump model: AUC = 0.81; *p* < 0.001. Loin model: AUC = 0.75; *p* < 0.001. Flank, rib, and forequarter model: AUC = 0.77; *p* < 0.001.

**Table 10:** Odds ratios and model metrics from logistic regression for bruises in beef cattle carcasses by age class across different primal cuts.

Age ^1^	Round and Rump			Loin			Flank, Rib, and Forequarter		
	Odds	LCI	UCI	Odds	LCI	UCI	Odds	LCI	UCI
Age 8-PIT (ref.)									
Intercept	0.68	0.665	0.686	0.12	0.122	0.125	0.49	0.485	0.499
Age 0-PIT	0.25	0.251	0.254	0.41	0.409	0.415	0.39	0.389	0.393
Age 2-PIT	0.30	0.301	0.305	0.46	0.460	0.465	0.42	0.421	0.424
Age 4-PIT	0.36	0.358	0.361	0.51	0.507	0.513	0.46	0.457	0.461
Age 6-PIT	0.50	0.501	0.505	0.62	0.614	0.623	0.58	0.575	0.579

Abbreviations: LCI = lower confidence interval; UCI = upper confidence interval; ref. = reference; PIT = permanent incisor teeth; AUC = area under the receiver operating. ^1^ Animals are categorized by dentition on the basis of age: ≤19 months = 0-PIT; 20–26 months = 2-PIT; 27–36 months = 4-PIT; 37–63 months = 6-PIT; and >63 months = 8-PIT. Model performance metrics: AUC and *p*-value: round and rump model: AUC = 0.82; *p* < 0.001. Loin model: AUC = 0.76; *p* < 0.001. Flank, rib, and forequarter model: AUC = 0.78; *p* < 0.001.

**Table 11:** Odds ratios and model metrics from logistic regression for bruises in beef cattle carcasses by sex across different primal cuts.

Sex	Round and Rump			Loin			Flank, Rib, and Forequarter		
	Odds	LCI	UCI	Odds	LCI	UCI	Odds	LCI	UCI
Females (ref.)									
Intercept	0.61	0.597	0.616	0.12	0.116	0.119	0.52	0.513	0.527
Beef bulls	0.23	0.233	0.235	0.35	0.348	0.352	0.25	0.249	0.251
Steers	0.49	0.488	0.494	0.71	0.706	0.718	0.47	0.463	0.469

Abbreviations: LCI = lower confidence interval; UCI = upper confidence interval; ref. = reference; AUC = area under the receiver operating. Model performance metrics: AUC and *p*-value: round and rump model: AUC = 0.82; *p* < 0.001. Loin model: AUC = 0.76; *p* < 0.001. Flank, rib, and forequarter model: AUC = 0.79; *p* < 0.001.

**Table 12:** Odds ratios and model metrics from logistic regression for bruises in beef cattle carcasses by subcutaneous fat score across different primal cuts.

Fat Score ^1^	Round and Rump			Loin			Flank, Rib, and Forequarter		
	Odds	LCI	UCI	Odds	LCI	UCI	Odds	LCI	UCI
FS-3 (ref.)									
Intercept	0.40	0.393	0.406	0.10	0.096	0.100	0.35	0.345	0.355
FS-1	0.40	0.399	0.405	0.32	0.316	0.324	0.41	0.407	0.413
FS-2	0.78	0.782	0.787	0.74	0.738	0.744	0.78	0.777	0.781

Abbreviations: LCI = lower confidence interval; UCI = upper confidence interval; ref. = reference; FS = fat score; AUC = area under the receiver operating. ^1^ FS-1 = absent fat; FS-2 = scarce fat; FS-3 = medium fat. Model performance metrics: AUC and *p*-value: round and rump model: AUC = 0.81; *p* < 0.001. Loin model: AUC = 0.76; *p* < 0.001. Flank, rib, and forequarter model: AUC = 0.77; *p* < 0.001.

## Data Availability

The raw data and statistical analysis scripts that support the findings of this study will be made available in FigShare at [10.6084/m9.figshare.28553846] during the peer-review process.

## Supplementary Materials

The following supporting information can be downloaded at: https://www.mdpi.com/article/10.3390/ani15172608/s1, Table S1: Total number of beef cattle carcasses and percentage with at least one bruise by Brazilian state; Figure S1: Spatial distribution of freight orders and bruise prevalence in beef cattle carcasses.
