# Supplementary material for: Risk Factors Associated with Bruises in Beef Cattle Carcasses
Source: Animals (Basel). 2025 Sep 5;15(17):2608. doi: 10.3390/ani15172608 (PMC12427533; doi:10.3390/ani15172608)
Supplement: Supplementary file 1 [file animals-15-02608-s001.zip › Table S1.pdf]

Table S1. Total number of beef cattle carcasses and percentage with at least one bruise by Brazilian state.

State	Animal count	Bruised carcasses (%)	Bruised primal cut (%)					
			Round	Rump	Loin	Flank	Rib	Forequarter
AC	411,082	10.79	3.04	1.59	2.14	7.15	1.50	0.59
BA	311,404	18.89	8.18	11.91	10.10	9.46	5.37	5.75
GO	2,548,291	19.69	8.46	4.79	4.43	6.66	7.42	4.76
MG	1,139,588	42.70	17.63	24.53	11.39	14.67	14.48	11.73
MS	4,091,492	31.98	13.57	14.99	7.59	17.71	8.18	6.09
MT	5,795,205	34.25	16.71	13.94	7.33	15.71	10.23	4.50
PA	1,224,177	66.45	52.89	28.33	14.53	32.93	22.56	20.28
RO	2,099,977	40.13	16.91	20.44	13.13	22.87	11.29	6.57
SP	1,122,079	25.96	4.98	5.56	4.78	13.38	4.72	7.52
TO	660,089	35.33	19.61	9.83	11.01	14.78	8.68	6.07

Abbreviations: AC = Acre; BA = Bahia; GO = Goiás; MG = Minas Gerais; MS = Mato Grosso do Sul; MT = Mato Grosso; PA = Pará; RO = Rondônia; SP = São Paulo; TO = Tocantins.
