# Supplementary figures and images for: Risk Factors Associated with Bruises in Beef Cattle Carcasses
Source: Animals (Basel). 2025 Sep 5;15(17):2608. doi: 10.3390/ani15172608 (PMC12427533; doi:10.3390/ani15172608)

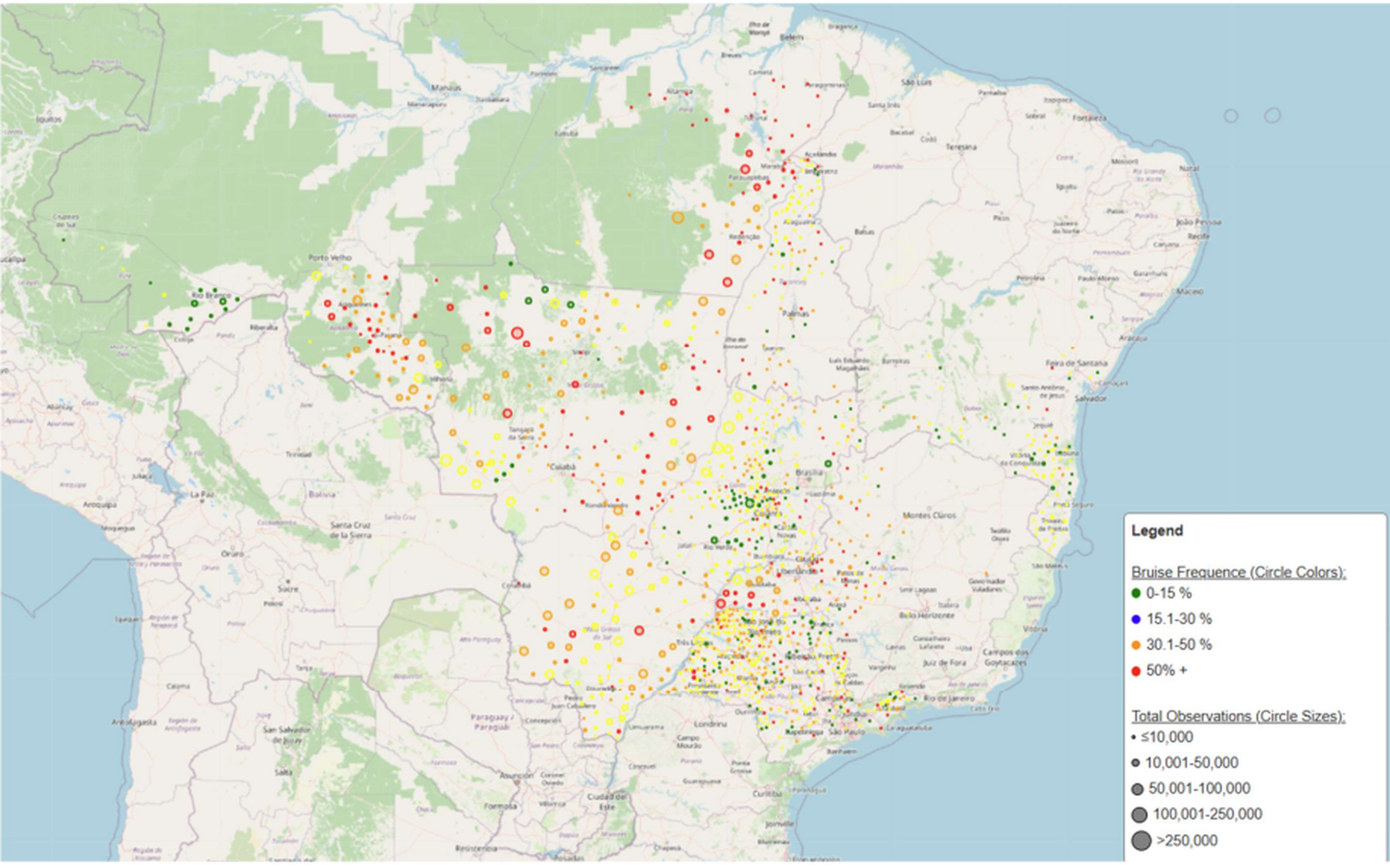

Supplement: Supplementary file 1 [file animals-15-02608-s001.zip › Figure S1.tiff]
